# Modes of Communication between T Cells and Relevance for Immune Responses

**DOI:** 10.3390/ijms21082674

**Published:** 2020-04-11

**Authors:** Lion F. K. Uhl, Audrey Gérard

**Affiliations:** The Kennedy Institute of Rheumatology, University of Oxford, Oxford OX3 7FY, UK; lion.uhl@gtc.ox.ac.uk

**Keywords:** T cells, communication, cytokines, synapses, integrins

## Abstract

T cells are essential mediators of the adaptive immune system, which constantly patrol the body in search for invading pathogens. During an infection, T cells that recognise the pathogen are recruited, expand and differentiate into subtypes tailored to the infection. In addition, they differentiate into subsets required for short and long-term control of the pathogen, i.e., effector or memory. T cells have a remarkable degree of plasticity and heterogeneity in their response, however, their overall response to a given infection is consistent and robust. Much research has focused on how individual T cells are activated and programmed. However, in order to achieve a critical level of population-wide reproducibility and robustness, neighbouring cells and surrounding tissues have to provide or amplify relevant signals to tune the overall response accordingly. The characteristics of the immune response—stochastic on the individual cell level, robust on the global level—necessitate coordinated responses on a system-wide level, which facilitates the control of pathogens, while maintaining self-tolerance. This global coordination can only be achieved by constant cellular communication between responding cells, and faults in this intercellular crosstalk can potentially lead to immunopathology or autoimmunity. In this review, we will discuss how T cells mount a global, collective response, by describing the modes of T cell-T cell (T-T) communication they use and highlighting their physiological relevance in programming and controlling the T cell response.

## 1. Introduction

T cells need to balance effective protection against pathogens with the risk of overreacting against themselves. In order to avoid immunologically driven tissue damage, T cells need to scale their clonal expansion and effector response according to the dose and strength of the antigenic trigger, which is accomplished by the temporal integration of T cell receptor (TCR) signalling with a number of additional regulatory signals [1]. The priming of T cells (reviewed here [2,3,4,5]) results in a population of individually highly heterogenous cells, which nevertheless respond as a collective, in a reproducible and integrated manner [6]. Considerable work has focused on understanding the various intrinsic and extrinsic cues driving this stochastic diversification process [3,4,7,8,9,10], but what often gets neglected is the fact that T cells never act on their own. Understanding T cell behaviour as highly inter-dependent and governed by population-level interactions may offer valuable clues. The control of immune cell activation, differentiation and expansion is primarily determined by a competition for trophic survival factors and other limiting factors, but the benefits of cooperative actions may often outweigh the benefits of each cell acting autonomously [11,12,13,14]. Coordination between T cells on a system-wide level relies on efficient and constant T-T communication. While the characterisation of T-T communication is still in its infancy, a list of potential mediators between CD8^+^ T cells has been recently identified through a bioinformatics approach, based on the expression of receptor-ligand pairs in T cells, their upregulation upon T cell activation, and their co-regulation in multiple infection models [15]. Overall, it is believed that various modes of short- and long-range intercellular communication act in concert to facilitate the coordination of individual cell actions into a collective response and will be reviewed in the next chapters. 

## 2. Modes of T Cell-T Cell Communication

CD8^+^ T cells are capable of proliferating and differentiating autonomously after antigen stimulation [16,17], suggesting that cell-cell communication and interactions beyond T cell- antigen-presenting cells (APC) interactions are crucial in shaping the exact outcome of the T cell response [18]. Direct T-T communication has been shown to modulate the differentiation of T cells [19,20,21,22,23]. Some communications happen over longer distances through the secretion and diffusion of soluble mediators such as cytokines and chemokines, while others require T cells to come in close contact. Together, the various modes of intercellular crosstalk facilitate T cells, to not only act as individual responders, but as a collective by coordinating their response. 

### 2.1. Long-Range T Cell-T Cell Communication

T cells produce and secrete soluble factors into the local environment, which enables them to translate and coordinate local interactions into tissue-wide global behaviour. Communication over long distances enables a population of immune cells to coordinate their individual behaviours and tune the global response towards an optimal outcome for the system. While few factors have been clearly established as mediating T-T cell communication, it is conceivable that others which are produced and consumed by T cells may also be implicated in T-T crosstalk. 

#### 2.1.1. a-Cytokines

Cytokine penetration in tissues is dependent on the density of local cytokine consumers, which contributes to non-genetic cell-to-cell variability by creating variable cytokine niches in the reactive microenvironment [24]. The current dogma is that cytokine penetration occurs when high levels of cytokines overwhelm the consumption capabilities of local consumers and thus lead to systemic diffusion throughout the tissue. 

One of the most studied cytokines involved in T-T communication is Interleukin (IL)-2 (IL-2). It has been discovered more than 30 years ago as a potent stimulator of T cell growth [25,26] and one of the most important regulators of the T cell response [27,28]. It is produced mainly by T cells [29] and promotes the maintenance of regulatory T (Treg) cells and the differentiation of CD4^+^ T cells into their effector subsets. For CD8^+^ T cells, IL-2 signals have been shown to optimize both effector T cell generation and memory differentiation [30,31]. While not being an absolute requirement for T cell proliferation and expansion, IL-2 has been proven by a number of studies to have a critical impact on the quality of the T cell response [28,32]. Strong IL-2 signals drive both CD4^+^ and CD8^+^ T cells to become terminally differentiated effector T cells, while a low-level of IL-2 signals drive responding CD4^+^ T cells towards the follicular helper T cell (TFH) or central memory state, and allow CD8^+^ T cells to survive as long-lived memory T cells [31].

Multiple inflammatory cytokines are shared between T cells. Activated CD4^+^ T cells differentiate into specific subtypes, producing a characteristic cytokine profile, depending mainly on the cytokine milieu of the microenvironment [33]. CD4^+^ T cells differentiating into T helper (Th) 1 (Th1) produce Interferon-γ (IFN-γ), IL-2, and tumour necrosis factor (TNF)-β. IFN-γ is a highly pleotropic cytokine associated with proinflammatory processes, but it also displays regulatory functions [34,35,36,37,38]. IFN-γ production by CD4^+^ T cells is sufficient to potentiate the role of IL-12 in promoting Th1 differentiation [20], while inhibiting Th17 differentiation [39]. IFN-γ produced by CD8^+^ T cells also skews their subsequent differentiation [21,35]. CD4^+^ T cells differentiating into Th2 produce IL-4, IL-5, IL-10, and IL-13. IL-4 is a signature cytokine in type II inflammatory responses and thus involved in the immune response against invading parasites and allergens. IL-4 produced by T cells [40,41] promotes Th2 differentiation, sustained in an autocrine/paracrine manner [42,43]. Both IL-4 and IFN-γ secretion by CD4^+^ T cells results in the ubiquitous phosphorylation of STAT6 or STAT1 in immune cells throughout reactive lymph nodes following *H. polygyrus*, *T. gondii* and *S. mansoni* infection, indicating that cytokines can function in a systemic manner under certain circumstances [44]. IL-6 is a proinflammatory cytokine produced by various immune cells, most notably dendritic cells and macrophages in response to inflammation, but is also expressed by subsets of activating T cells [45,46,47], and could potentially mediate T cell communication. It is known to promote Th17 development [48], while inhibiting the differentiation of Foxp3+ Treg cells [49].

Anti-inflammatory cytokines are also used by T cells to co-regulate their responses. Transforming growth factor β (TGF-β) is a pleiotropic cytokine that plays an essential role in T cell development, homeostasis, tolerance, and immune responses [50,51,52,53,54]. It is produced by many different cell types, including activated CD4^+^ T cells [55] and Treg cells [56]. TGF-β secretion by Treg cells plays an important role in inhibiting T cell proliferation, activation, and differentiation [57], and controlling the expansion of short-lived effector CD8^+^ T cells, by promoting their apoptosis during clonal expansion [58]. Interestingly, TGF-β is stored in the extracellular matrix as a latent complex and its activation requires the binding of αv integrin [59]. As a consequence, TGF-β acts locally and specifically, while being produced more systemically [50]. IL-10 is a well-studied immunosuppressive cytokine, that has been shown to limit IFN-γ production and control autoimmune inflammation [60,61,62]. It is known to be produced by different immune cell types, including Th1, Th2, Th17, Treg and CD8^+^ T cells [63], suggesting that IL-10 could be used by T cells to co-regulate each other. 

#### 2.1.2. b-Chemokines 

Chemokines are chemotactic cytokines orchestrating the migration and positioning of all immune cells and are critically involved in diverse immune cell processes, such as cell fate or activation [64]. In vivo, activated CD4^+^ T cells clustering around DCs cause CD8^+^ T cell accumulation through CCL3/CCL4-CCR5 binding, which enhances CD8^+^ T cell/DC contact formation [65], highlighting the crucial role of chemokines in enabling activating T cells to find each other and create the niche that serves as a platform for subsequent T-T communication. While this mechanism relies on another cellular intermediate, it is conceivable that T cells also directly attract each other via other chemokines, as they up-regulate multiple chemokine/chemokine receptor pairs [15].

#### 2.1.3. c-Exosomes

Immune cells are also known to communicate through the secretion of extracellular vesicles, in particular endosomally derived exosomes [66]. Exosomes are capable of carrying a number of different molecules, which makes them ideal conduits for the intercellular transfer of information. The transfer of genetic information in secreted exosomes such as messenger RNA (mRNA) and microRNA (miRNA) suggests that exosomes are also utilized to regulate the protein expression of target cells [67,68,69]. Exosome secretion by T cells has been shown by a number of studies to be involved in the regulation of T cell responses [70,71,72]. However, most of the studies investigating exosome transfer between immune cells have been performed in vitro and the biological significance in vivo remains unclear. 

### 2.2. Contact-Based T Cell-T Cell Communication

#### 2.2.1. a. Synapse

An important feature of an effective immune response is the specific activation of appropriate responders and the subsequent directed targeting of an invading pathogen while sparing self, which necessitates excluding bystanders. In many cases this is accomplished by the formation of adhesive interfaces between the surfaces of interacting immune cells, with a synaptic space in between them. The immunological synapse (IS) formed between T cells and APCs is the best studied example of such a structure [73]; but other types of immune synapses, such as T-T synapses, have also been described [19,22]. 

**Figure 1:**
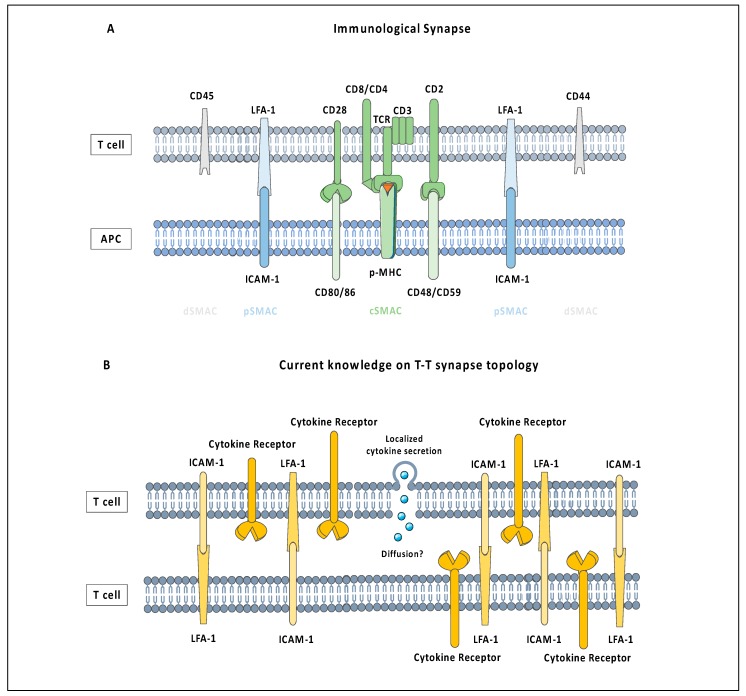
Comparison between the immunological synapse formed between an antigen-specific T cell and antigen-presenting cells (APC) (**A**) and the T-T cell synapse (**B**). While the T-APC immunological synapse is characterized by a high level of membrane organization, the T-T synapse does not show a clear structural organization. Cytokines are secreted at the centre of T-T synapses, but it is unclear if they diffuse into the cleft between the cells or are immediately taken up by receptors, which have been observed to be enriched at contact sites, but without being organized into distinct clusters.

Immunological synapses between antigen-specific T cells and APCs are sites of signal initiation and integration for T cell activation and subsequent programming [74,75,76]. It is characterized by the formation of a ‘bullseye’ pattern, consisting of a central supramolecular activation cluster (cSMAC), which is surrounded by a peripheral supramolecular activation cluster (pSMAC) (Figure 1A). While the cSMAC consists mainly of clustering TCRs, cross-junctionally interacting with pMHC, the pSMAC is rich in adhesion molecules such as leukocyte function-associated antigen-1 (LFA-1), which serve to stabilize long-term cell-cell interactions. Several other molecules, such as CD2, CD4, CD8, CD28, Lck and Fyn have also been identified to predominantly cluster in this peripheral region [77]. 

Antigen-specific T cells also interact with themselves, forming clusters during priming in secondary lymphoid organs (SLOs) [22,78,79,80]. T cell clusters are a hallmark of efficient T cell activation in in vitro cultures. Similar to T-APC contacts, T-T interactions are mediated by the activated form of LFA-1, which binds to its ligands intercellular adhesion molecule (ICAM), ICAM-1 (also known as CD54) being the most studied [81,82,83,84]. LFA-1, ICAM-1 and ICAM-3 are constitutively expressed in T cells, irrespective of their phenotype, and ICAM-1 expression is strongly upregulated upon T cell activation [85,86,87]. In order to bind to its ligands, LFA-1 needs to transition from a closed to an active conformation, which is induced by TCR triggering. As a result, only primed T cells engage in T-T contacts [83,87]. Electron microscopy revealed that contacts between responding CD4^+^ T cells are organized in multifocal synapses [22]. Modest enrichment of CD86, MHC, TCR, CD4, and CD28 is found within the T-T synapse (Figure 1B), which contrasts the organization of the IS formed between T cells and APCs [22]. 

T-T interactions are fairly dynamic, with dwell times of around 5−10 min and thus much shorter than the interaction times between T cells and APCs [19]. Despite their transient nature, these structures facilitate the directional sharing of the cytokines IL-2 and IFN-γ between interacting T cells [19,21,22]. In addition, it provides a platform for co-incidental signalling between different mediators. For instance, the receptor of IFN-γ colocalizes at T-T synapses with ICAM-1 and LFA-1 [21]. This results in the integration of both downstream signalling pathways at the T-T synapse, where STAT1 phosphorylation on Tyrosine 701 triggered by IFN-γ signalling is enhanced by integrin-mediated activation of the Src kinases Fyn or Lck [21]. This suggests that ICAM-1 and LFA-1 can function not only as adhesive structures, but also as co-stimulatory molecules at the T-T synapses. This was first highlighted by the fact that the antibody-mediated ligation of ICAM-1 on human T cells has a costimulatory effect similar to anti-CD28 antibody-mediated co-stimulation, by enhancing CD3-mediated T cell activation [88]. While LFA-1 is believed to be the primary adhesion molecule mediating T-T interactions, other adhesion molecules, such as members of the SLAM family, have also been shown to promote T cell clustering [89]. This suggests that T-T synapses are platforms which foster the integration of a diverse mix of signals shared exclusively between T cells. 

#### 2.2.2. b. Tunnelling Nanotubes

Another contact-mediated mode of communication between T cells which is not restricted to immediately adjacent cells is created through the formation of so called tunnelling nanotubes (TNTs) [66,90]. These structures, consisting of long thin F-actin-based membranous channels connecting cells, have been described in numerous cell types including almost all immune cells [91]. While it has been shown that TNTs enable the transfer of both soluble signals and cargo such as mitochondria or other organelles, it proved difficult to clearly elucidate the functional significance of these structures during an immune response, especially in vivo. Nevertheless, while many open questions remain, TNTs open up interesting possibilities for intercellular communication, by facilitating the sharing of a wide range of different biological material between cells [66].

## 3. Relevance of T Cell-T Cell Communication

### 3.1. Regulation of T Cell Priming 

Infections with virus may generate less than 100 APCs per lymph node [92], and an early protective response is crucial to limit dissemination. In addition, the number of T cells specific for a given antigen is about one in 10^5^–10^6^ T cells [93]. In order to behave collectively, T cells have to overcome these limiting factors and develop strategies to communicate despite the unlikelihood of being in close proximity. Various studies have demonstrated that activated T cells are able to find each other [65,94,95]. Multiple chemokine/receptor pairs are upregulated by T cells following priming [15], suggesting that primed T cells can attract each other. Similarly, antigen-specific CD8^+^ T cells favour the priming of naive CD8^+^ T cells, by promoting the CCR5-dependent recruitment of polyclonal CD8^+^ T cells to mature dendritic cells [94], resulting in the amplification of priming. Another strategy to regulate T cell priming relies on the acquisition of pMHC complexes from APCs by T cells through trogocytosis. While it was suggested to be important in downregulating the response by tagging activating T cells for peptide-specific lysis by neighbouring T cells [96], other studies showed that it endows T cells with some of the functional properties of APCs and thus additional regulatory capabilities, which can have both stimulatory or suppressive effects on the ensuing immune response [97,98,99,100,101,102,103,104,105]. Helft et al. has observed that pMHC acquisition from APCs by CD4^+^ T cells and subsequent presentation of these complexes to other CD4^+^ T cells is used as a mechanism by T cells to inhibit the recruitment of antigen-experienced CD4^+^ T cells into the response, favouring the recruitment of naïve T cells [106].

### 3.2. Sensing Population Size During Immune Responses

T cells have been shown to use mechanisms similar to quorum sensing to regulate their population size. Quorum sensing is a type of cellular communication mediated by extracellular factors generally produced and detected by the same type of cells, referred to as autoinducers. The detection of autoinducers and the activation of downstream signalling pathways occurs upon reaching a sufficiently high autoinducer concentration within the local environment, which in turn requires a sufficiently high cell population density of signal producers [107]. While many of these discoveries may be specific for the microbial world [108], there is evidence that similar mechanisms also act amongst immune cells to control their responses [109,110]. 

The cytokine IL-2 has been suggested as functioning as an autoinducer-like molecule between T cells [12,32]. In vitro, a minimal number of IL-2 secreting effector CD4^+^ T cells is required to maintain critical levels of phosphorylated signal transducer and activator of transcription 5 (STAT5) in CD4^+^ T cell, which are necessary for T cell activation and subsequent expansion, indicating a population density-dependent regulation [111]. Savir et al. generated a mathematical model to analyse the secrete-and-sense circuit in T cells [112]. Their model demonstrated that a group of T cells can compete for the same global pool of secreted IL-2 by tuning their ‘activation threshold’ (the IL-2 concentration at which cells can switch on proliferation-promoting genes). Within a population of polyclonal T cells with distinct activation thresholds, cells are consequently both capable of either competing or cooperating with each other, thus leading either to autocrine or a form of paracrine communication resembling quorum sensing. Antigen-specific CD8^+^ T cells similarly rely on autocrine IL-2 production by neighbouring activating CD8^+^ T cells as the main source of this critical cue, rather than depending on paracrine IL-2 signals from CD4^+^ T cells or DCs [31,113]. Recently, a paper by Zenke et al. demonstrated that nested antagonistic IL-2 and CD80/86-CTLA-4-mediated feedback mechanisms function to promote or inhibit CD8^+^ T cell expansion, based on cellular density [15]. ICAM-1-mediated cell clustering facilitates T cell population-intrinsic mechanisms, which enable T cells to collectively self-regulate their proliferation and apoptosis during their expansion. Interestingly, IL-2 exerts seemingly paradoxical effects depending on its concentration in the local environment. Using well-controlled in vitro experiments and mathematical models, Hart et al. demonstrated that IL-2 mainly promotes cell death above a critical local population density of IL-2-secreting cells, while acting pro-proliferative if cell numbers are below this threshold concentration [114], suggesting that the specific effects of IL-2 on cells are cell density-dependent, a feature of quorum sensing. 

Both IL-2 receptor and cytokine expression are controlled by a number of feedback mechanisms [111]. Under steady state, naïve CD4^+^ T cells do not express IL-2 and IL-2Rα (CD25) at significant levels, but production of both proteins is strongly induced upon activation by antigen. Because IL-2Rα levels remain low at the beginning of the immune response, and increase around 2 days after priming, IL-2 can accumulate in the environment without cells immediately responding to this signal (a classic feature of an autoinducer-like signalling factor). Once the receptor levels reach a critical threshold, IL-2 signalling in CD4^+^ T cells is induced, leading to the activation of two feedback loops—a positive feedback loop driving further IL-2Rα expression and eventually a negative feedback loop inhibiting the production of more IL-2 [115]. 

### 3.3. Regulation of T Cell Differentiation

T cell differentiation is a highly complex process, and varying models have been proposed to explain the fate determination of responding cells [4], but T collective behaviour is emerging as an underappreciated parameter controlling differentiation at the population level.

During TCR activation in a particular cytokine milieu tailored to the invading pathogen, naive CD4^+^ T cells may differentiate into one of several subtypes of T helper (Th) cells, including Th1, Th2, Th17, and iTreg [33]. CD4^+^ T cells are capable of autonomously modulating their differentiation towards Th1 fate, in response to infection through secretion of IFN-γ [20]. In Th1 cells, the transcription factor T-bet, which is markedly induced by IFN-γ [116], induces Runx3 expression, which synergizes with T-bet to promote a feedforward loop to enhance IFN-γ expression, while supressing the expression of the Th2-fate program-inducing cytokine IL-4 [117,118]. Th1 cells also downregulate IFN-γ receptor 1 (IFN-γR1), which results in Th1 cells becoming insensitive to IFN-γ-induced apoptosis [119]. Given the observation that cytokine expression in CD4^+^ T cells is stochastic [120,121], and thus characterized by only a small subset of cytokine producers, it seems reasonable to assume that these effects may also be regulated by local density. 

The T cell response to infection has two primary goals. The first one is to fight an invading pathogen and present infection, by the generation of a large number of effector cells. The second goal is to retain a smaller subset of responders with enhanced longevity and regenerative capacity, to protect against future encounters with the same pathogen. This is achieved by the expansion of T cells upon activation and the subsequent differentiation into effector and memory subsets. A recent study by Polonsky et al. indicated that the differentiation of CD4^+^ T cells in effector and memory subsets is regulated by local cell density [122]. Increased cell density drives the differentiation of naïve CD4^+^ T cells into memory precursor cells. The authors identified the cytokines IL-2 and IL-6 as the main drivers of collective CD4^+^ T cell memory differentiation, with IL-2 regulating the proliferation rate and IL-6 promoting the survival of responding T cells. Blocking both cytokines resulted in a reduction of CD62L expression, a phenotypic marker of memory T cells. The cell surface molecule SLAMF6 was also to be involved, by causing a reduction of CD62L expression on responding T cells. 

Multiple papers also implicated the requirement of IFN-γ for effector and memory differentiation of CD8^+^ T cells [123,124], some showing it directly acts through T-T communication [19,21,35,36,37].

A number of groups investigated how homotypic clusters shape the differentiation of T cells. In CD4^+^ T cells, LFA-1/ICAM-1 ligation in human T cells promotes Th1 polarization [125]. T-T interactions have also been shown in vitro to trigger CD4^+^ T cell activation, proliferation, and differentiation [126], and in vivo to induce the generation of CD4^+^ T cells with a regulatory phenotype, characterised by the production of IL-4, IL-10 and TGF-β [85]. In CD8^+^ T cells, LFA-1/ICAM-1 dependent clustering promotes their expansion [15,19,23]. Mechanistically, cluster dependent-expansion is linked to CTLA-4 and CD80, which mediate activation of the Hippo pathway, linking the magnitude of clonal expansion by activated cells to their commitment to terminal differentiation, through the induction of Blimp1 [127]. However, the function of LFA-1/ICAM-1 interactions in CD8^+^ T cell differentiation and function is unclear, as blocking CD8^+^ T cell clustering has been shown to increase effector differentiation and IFN-γ production in vitro and following Incomplete Freund Adjuvant (IFA)-OVA priming [23], while it decreases effector differentiation [15,19] and IFN-γ production in vivo, following multiple infection and vaccination models [19]. Although the reason for those discrepancies is unclear, it is tempting to speculate that T cell cluster formation through LFA-1/ICAM-1 interactions constitute a platform where different signals are integrated, and the outcome is tailored to the injury and the type of messages shared in clusters.

Finally, evidences suggest that T cell contacts happen between different T cell subsets. Direct contacts between antigen experienced CD8^+^ memory T cells and naïve CD8^+^ T cells during priming promote the accelerated differentiation of naïve cells into effector memory (TEM) cells, at the expense of less differentiated stem cell memory (TSCM) and central memory (TCM) cells [128]. These effects are mediated by contact-dependent nonapoptotic Fas-FasL interactions. The extent of accelerated naïve CD8^+^ T cell differentiation was dependent on the density of the two subsets, suggesting again a quorum sensing-like regulatory mechanism. 

### 3.4. Regulation of Peripheral Tolerance

T cells are fairly cross-reactive and occasionally autoreactive, even in healthy individuals. The control of autoimmune T cells requires a number of stringent regulatory mechanisms, to avoid detrimental reactions and a breach of self-tolerance [129,130]. Studies have suggested that population-wide decisions made by T cells act as important safeguards in ensuring reliable responsiveness to pathogens, while controlling each individual T cell’s potential for autoimmunity [131]. As such, the relevance of controlling the response on a population-wide level may not only lie in ensuring optimal protection against a pathogen, but also in avoiding autoimmunity.

At steady state, IL-2 drives the control of auto-reactive T cells. Treg cells, which are critically involved in maintaining self-tolerance [57], require IL-2 for their survival and expansion [132,133,134], but they cannot produce IL-2 themselves, due to the repression of the *il2* gene by FOXP3 [135,136]. At steady state, CD4^+^ T cells are the main producers of IL-2 [12,134,137,138], and the main source of IL-2 for maintaining a fully functional Treg cell population [134]. A study by Liu et al. observed, using in vivo live imaging, that a large number of potentially autoimmune CD4^+^ T cells get constantly activated by self-peptides, which results in IL-2 production and the subsequent recruitment and suppression by Treg cells, to prevent the expansion of autoimmune CD4^+^ T cells [139]. 

During an immune response, communication mediated by IL-2 during priming can both lead to cooperation or competition between effector CD4^+^ T cells [31,112,140,141,142] and the inhibition of effector T cells by Treg cells [32,111]. This complex interplay between immune activation and suppression requires a number of finely tuned regulatory feedback mechanisms, which have been suggested by a number of groups to be quorum sensing-like [12,143]. A threshold number of locally activated, IL-2-secreting, T cells is required to escape Treg-mediated immune suppression and result in successful activating T cell proliferation. Quorum sensing thus imposes a population consensus on the immune response, which ensures an appropriate balance between immunity and tolerance [143], a hypothesis which has been further developed by different groups over the years to incorporate novel insights about the biology of Treg cells and IL-2 [144]. Of note, Treg cells have also been implicated in the IL-2-mediated fine-tuning of the CD8^+^ T cell responses by limiting IL-2 production and thus shaping the differentiation of the responding cells [145,146,147]. 

Finally, T-T cell contacts were also suggested to enhance Treg cell-mediated suppression by potentiating IL-35 and IL-10 production [148], suggesting another mechanism by which T cells control tolerance. 

### 3.5. Regulation of Effector Responses

Regulatory interplay between T cells has recently been observed at peripheral sites. Treg cells in the tumour microenvironment have been shown to restrain the IL-2-dependent acquisition of cytotoxic functions by CD4^+^ T cells, impeding anti-tumour immunity [149]. Interleukin-35 (IL-35), a member of the IL-12 cytokine family, is produced by Treg cells in tumours, where it promotes CD4^+^ and CD8^+^ T cell exhaustion [150]. T cell clustering also contributes to enhanced T cell retention in tumours, by entrapping activated CD8^+^ T cells in the tumour microenvironment [151]. While not being the focus of this study, it is reasonable to assume that beyond better retention adhesive contacts between T cells in this context may again facilitate T cell-mediated regulation of the response by directional cytokine sharing. 

T cell clustering was also observed in other anatomical sites, such as the liver [152]. Priming by Kupffer cells leads to the formation of dense, extravascular clusters of immotile cells and the development of effector cells. By contrast, priming by hepatocytes leads to loose, intravascular clusters of motile cells and the development of dysfunctional cells. The observation that both priming mechanisms resulted in qualitatively different T cell clusters suggests that homotypic T cell interactions may play a critical role in delivering diverse signalling cues tailored to the injury. In addition, in a murine model of hepatitis B, IL-10 produced by activated CD8^+^ T cells upon hepatocellular antigen recognition acts in an autocrine/paracrine fashion to increase IL-2 responsiveness and rescue CD8^+^ T cells from antigen-induced apoptosis [153].

Intercellular T-T communication can also be mediated by the intercellular transfer of membrane components, through a process known as trogocytosis. The sharing of TCRs between cytotoxic T lymphocytes through trogocytosis has been reported [154]. Antigen-specific T cells are rare and in order to mediate a successful immune response, they need to rapidly expand upon activation. The transfer of TCRs to recipient cytotoxic T cells of an unrelated specificity increases the numbers of responding cells and may enable T cells to amplify their response if necessary.

## 4. Outlook

**Figure 2:**
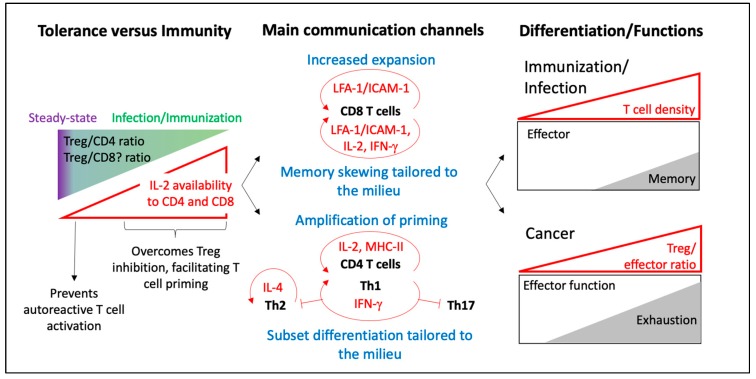
Current knowledge on T-T communication and associated functions. Cartoon summarizes the involvement of T-T communication in the balance between tolerance and immunity, the main communication mechanisms they use and the consequences during Infection and Cancer. Features of T cell communication are shown in red, and the outcome of T cell communication is shown in blue. At steady state, auto-reactive T cells are kept at bay, because of a high Treg/T cell ratio. Following infection, this ratio decreases, resulting in T cell priming through increased IL-2 availability (**left panel**). Following activation, T cells use a variety of communication channels to regulate their expansion and differentiation required and according to the injury (**middle panel**). This results in a robust population response following infection, fine-tuned by T cell density. T cell communication can be co-opted in cancer, promoting T cell exhaustion (**right panel**).

It is becoming increasingly clear that T cell communication is central to coordinate T cell responses and tailor it to the injury while preserving tolerance (Figure 2). Some mediators have been described to mediate this communication (Figure 2), but it seems likely that other molecular players are involved. In addition, it remains to be determined how collaboration and competition for niche and trophic factors work together in orchestrating the immune response and if a specific microenvironment or particular context favours one over the other. While cytokines are the best studied examples, other molecules such as metabolites or even exosomes may possibly also function as autoinducer-like factors. What is certain is that T cells use a vast repertoire of communication mechanisms and a better understanding of the T cell response will require further understanding of how these different components work together.

Intercellular communication between T cells has been firmly established to be a crucial regulator of the T cell response, by modulating their activation, expansion and differentiation. It has also been implied to be involved avoiding autoimmunity, by balancing the conflicting demands of self-tolerance and responsiveness to a diverse range of pathogens [131]. Given that cytokine secretion profiles at the single cell level is quite heterogeneous, the cooperation of activating T cells through intercellular communication may be required to ensure optimal activation and differentiation.

Given its critical involvement in controlling T cell homeostasis, immune cell activation and population size, it is easy to see how the dysregulation of quorum sensing mechanisms may result in immunopathology or autoimmunity. A prototypic example of the result of a failure of regulatory mechanisms in maintaining homeostasis is cancer, and it is thus not surprising that disruption of quorum sensing has been implicated in tumorigenesis, which highlights the general relevance of this cellular communication mode in controlling correct cellular behaviour [155,156,157].
